# Increased Use of Hyperosmolar Therapy for Suspected Clinically Apparent Brain Injury in Pediatric Patients with Diabetic Ketoacidosis during the Peak of the COVID-19 Pandemic

**DOI:** 10.1155/2023/5123197

**Published:** 2023-02-28

**Authors:** Svetlana Azova, Enju Liu, Joseph Wolfsdorf

**Affiliations:** ^1^Division of Endocrinology, Boston Children's Hospital, Boston, Massachusetts 02115, USA; ^2^Department of Pediatrics, Harvard Medical School, Boston, Massachusetts 02115, USA; ^3^Institutional Centers for Clinical and Translational Research, Boston Children's Hospital, Boston, Massachusetts 02115, USA

## Abstract

The incidence of pediatric diabetic ketoacidosis (DKA) increased during the peak of the COVID-19 pandemic. The objective of this study was to investigate whether rates of hyperosmolar therapy administration for suspected clinically apparent brain injury (CABI) complicating DKA also increased during this period as compared to the three years immediately preceding the pandemic and to compare the characteristics of patients with suspected CABI before the pandemic, patients with suspected CABI during the peak of the pandemic, and those with DKA but without suspected CABI during the pandemic. Patients aged ≤18 years presenting with DKA before (March 11, 2017–March 10, 2020) and during the peak of the pandemic (March 11, 2020–March 10, 2021) were identified through a rigorous search of two databases. Predefined criteria were used to diagnose suspected CABI. Biochemical, clinical, and sociodemographic data were collected from a comprehensive review of the electronic medical record. The proportion of patients with DKA who received hyperosmolar therapy was significantly higher (*P* = 0.014) during the pandemic compared to the prepandemic period; however, this was only significant among patients with newly diagnosed diabetes. Both groups with suspected CABI had more severe acidosis, lower Glasgow Coma Scale scores, and longer hospital admissions (*P* < 0.001 for all) than cases without suspected CABI. During the pandemic, the blood urea nitrogen concentration was significantly higher in patients with suspected CABI than those without suspected CABI, suggesting they were more severely dehydrated. The clinical, biochemical, and sociodemographic characteristics of patients with suspected CABI were indistinguishable before and during the pandemic. In conclusion, administration of hyperosmolar therapy for suspected CABI was more common during the peak of the COVID-19 pandemic, possibly a result of delayed presentation, highlighting the need for increased awareness and early recognition of the signs and symptoms of diabetes and DKA, especially during future surges of highly transmissible infections.

## 1. Introduction

Numerous observational studies have shown that the incidence of pediatric diabetic ketoacidosis (DKA), and specifically severe DKA, increased during the time period corresponding to the peak of the COVID-19 pandemic [1–9]. There are sparse data, however, on the frequency of altered mental status or clinically apparent brain injury (CABI), and no significant differences have been reported between the prepandemic and pandemic time periods [3, 7]. Ho et al. found a trend toward significance in the number of children newly diagnosed with type 1 diabetes mellitus (T1DM) who received hyperosmolar therapy (hypertonic saline or mannitol) [3].

Based on anecdotal and observational experience, we explored whether the incidence of hyperosmolar therapy administration for suspected CABI complicating DKA in pediatric patients was higher during the peak of the COVID-19 pandemic as compared to the previous three years. We also collected biochemical, clinical, and sociodemographic data to determine how patients presenting with suspected CABI during the pandemic may have differed from patients presenting with suspected CABI prepandemic and those presenting in DKA but without suspected CABI during the pandemic. Recognition of an increased incidence of suspected CABI, indicative of more severe DKA, during the peak of the pandemic may have important implications for prognosis and counseling of these patients and may help inform future public health initiatives to promote prompt recognition of signs associated with new onset diabetes and DKA.

## 2. Materials and Methods

### 2.1. Study Design, Setting, and Selection of Participants

In this retrospective chart review study, we identified all children age ≤18 years who directly presented or were transferred to Boston Children's Hospital (BCH), a large quaternary referral center, with DKA in the three-year period before the COVID-19 pandemic (March 11, 2017–March 10, 2020) and during the one-year time period corresponding to the peak of the pandemic (March 11, 2020–March 10, 2021). This cutoff was based on when the World Health Organization officially declared the COVID-19 outbreak a pandemic (March 11, 2020). To identify the study subjects, we used the i2b2 tool to query BCH Healthcare electronic heath record data to find all the records that contained the term “ketoacidosis” during the prespecified time periods, as well as records that had the terms indicative of suspected CABI (“cerebral edema,” “brain injury,” “mannitol,” “3% sodium chloride,” “mechanical ventilation,” and “intubation”) appearing in the same encounter as the term “diabetes.” In addition, we reviewed the Inpatient Diabetes Quality Improvement Database, which maintains an up-to-date record of all patients with diabetes that present to BCH. We subsequently performed a meticulous review of the electronic medical records, cross-checking both databases to ensure completeness and accuracy, and identified all patients who presented with DKA between March 11, 2017 and March 10, 2021. We then identified all patients with DKA who received hyperosmolar therapy (either mannitol or hypertonic saline) for suspected CABI. In addition to calculating the proportion of cases who received hyperosmolar therapy for mental status changes before and during the pandemic, we also obtained and compared biochemical, clinical, and sociodemographic variables among three groups: suspected CABI patients before the pandemic (Group 1), suspected CABI patients during the pandemic (Group 2), and DKA cases without suspected CABI during the pandemic (Group 3). Ethics approval was granted by Boston Children's Hospital Institutional Review Board. A waiver of informed consent was obtained.

### 2.2. Definitions of Outcomes

DKA was defined using the International Society for Pediatric and Adolescent Diabetes 2018 guidelines: plasma glucose >200 mg/dL, venous pH < 7.3 or serum bicarbonate <15 mmol/L, and the presence of ketonemia (beta-hydroxybutyrate [BOHB] ≥3 mmol/L) or ketonuria (≥2+ or “moderate or large”) [10]. The diagnosis of suspected CABI was based on deterioration in neurologic status leading to initiation of hyperosmolar therapy or endotracheal intubation or radiologic evidence of cerebral edema [11].

### 2.3. Collection of Variables

The following biochemical variables on presentation were collected: venous pH, partial pressure of venous carbon dioxide (pCO2), plasma BOHB, serum bicarbonate, glucose, sodium, potassium, chloride, blood urea nitrogen (BUN), creatinine, and osmolality, and hemoglobin A1c (HbA1c). In the few cases where an initial arterial blood gas was obtained, we calculated venous pH as arterial pH – 0.05 and venous pCO2 as arterial pCO2 +6 mmHg [11]. If serum bicarbonate was not available, the bicarbonate concentration from a venous blood gas analysis was used. Corrected sodium was computed as (glucose − 100) × 0.016. Anion gap was calculated as sodium–(chloride + bicarbonate). Effective osmolality was calculated as (2 × sodium) + (glucose/18). To determine the change in HbA1c from baseline, a follow-up HbA1c was collected at least 12 months after the initial value obtained on presentation.

The following clinical variables were collected: date and time of presentation, transfer status, new diabetes diagnosis versus established diabetes, type of diabetes (type 1, type 2, or other), initial and lowest Glasgow Coma Scale (GCS) score, length of stay (in days), and presence of suspected or documented infection. For patients presenting with suspected CABI, the following additional variables were collected: neurologic status and results of the cranial computed tomography (CT) imaging, the time from presentation to the initiation of hyperosmolar therapy (as a surrogate for the time to development of suspected CABI), the time between the first and last hyperosmolar treatment (as a surrogate for duration of treatment), the number of hyperosmolar treatments, and patient outcome.

The following sociodemographic variables were collected: age at presentation, sex, race/ethnicity, insurance type, caregiver scores on the Newest Vital Sign health literacy assessment tool (expressed as an average for multiple caregiver scores) [12], caregiver occupations, presence of an intact family (defined as families in which both biological parents are present in the home), and presence of developmental/psychiatric concerns in the child. Caregiver occupations were assigned a skill level from 0–3 (0 if unemployed) based on a system modified from the International Standard Classification of Occupations (expressed as an average for multiple caregiver skill levels) [13]. As the caregiver occupation code was chosen as a surrogate for socioeconomic status, caregivers not working outside the home (e.g., stay-at-home parents and homemakers) were assigned a skill level of 0 if they did not have an income.

### 2.4. Statistical Analyses

The proportions of patients with suspected CABI out of all cases presenting with DKA were compared between the two prespecified time periods, before and during the pandemic, using the Pearson chi-square test. Stratified analyses by new diabetes diagnosis versus established diabetes, diabetes type, and the two factors combined were also performed using either the Pearson chi-square test or Fisher's exact test depending on cell counts. Continuous data were assessed for normality using the Shapiro‒Wilk test and for homogeneity of variances (based on mean) with Levene's test. For normally distributed data with equal variances across all groups, an independent two-sample *t*-test or one-way analysis of variance with Tukey post hoc test were used to evaluate for differences in means across groups. For non-normally distributed data in at least one group and and/or unequal variances across the groups, the Mann‒Whitney U test or Kruskal‒Wallis test with pairwise comparisons of mean ranks was utilized. For categorical variables, we used the Pearson chi-square test or Fisher–Freeman–Halton Exact Test depending on cell counts. Where appropriate, Bonferroni correction for multiple tests was performed for significant results. To compare change in HbA1c from baseline, a general linear regression model was constructed with baseline HbA1c, time interval between the two HbA1c measurements (in days), and new versus established diabetes status added as covariates. If unequal variances across groups were present, parameter estimates with robust standard errors were calculated. A two-sided *P* value of <0.05 was used to define statistical significance. Statistical Package for Social Sciences International Business Machines Corporation (IBM) software package (Version 28.0, IBM, Armonk, NY) was used for all statistical analyses.

## 3. Results and Discussion

### 3.1. Results

A total of 503 episodes of DKA occurred across the two combined time periods (344 before the COVID-19 pandemic, 159 during the peak of the pandemic). During the pandemic, 11.3% of patients received hyperosmolar therapy for mental status changes compared to 5.2% in the three-year period before the pandemic, with *χ*2 (1, *n* = 503) = 6.066 and *P* = 0.014 (Table 1). The proportion of patients presenting with new versus established diabetes did not differ significantly before and during the pandemic, with *χ*2 (1, *n* = 503) = 0.185, *P* = 0.691. The most common presumed causes of DKA in patients with established diabetes were nonadherence with insulin therapy and concern for pump malfunction. A few were thought to have symptoms of a viral infection; however, none of the patients with established diabetes who presented during the pandemic tested positive for COVID-19. Stratified analysis showed a statistically significant difference in the proportion of patients receiving hyperosmolar therapy exclusively in those with new onset diabetes, with *χ*^2^ (1, *n* = 319) = 4.751 and *P* = 0.029.

Of 503 episodes of DKA, 17 patients did not have T1DM (12 type 2 diabetes, 2 mitochondrial, 1 medication-induced, 1 post-transplant, and 1 post-subtotal pancreatectomy for congenital hyperinsulinism). In the prepandemic period, two patients with suspected type 2 diabetes (based on weight/body mass index >99th percentile for age and sex, presence of acanthosis nigricans, family history positive for type 2 diabetes, and negative pancreatic islet autoantibodies in both, as well as a normal baseline C-peptide level in one of the two patients in whom it was measured) presented with severe hyperosmolar DKA and initial serum glucose concentration >1500 mg/dL and were treated with mannitol due to mental status alterations but without notable improvement. Both patients had brain imaging that did not show evidence of cerebral edema. Because their mental status changes were subsequently attributed to hyperosmolality, these patients were not included in the prepandemic suspected CABI group.

Both Group 1 (suspected CABI patients before the pandemic) and Group 2 (suspected CABI patients during the peak of the pandemic) had significantly more severe acidosis on presentation compared to Group 3 (DKA cases without suspected CABI during the pandemic) (Table 2). The BUN concentration was higher in Group 2 compared to Group 3 but did not differ significantly between Groups 1 and 3. The BUN/creatinine ratio was significantly higher in Group 2 (28.5 ± 9.4) compared to the other two groups. Groups 1 and 2 did not differ from each other on any biochemical parameter (Table 2).

There were no statically significant differences among the groups in either the initial or follow-up HbA1c values; however, Group 2 showed a significant decrease in HbA1c from baseline as compared to Group 3 (*P* = 0.01) (Table 2).

Groups 1 and 2 had significantly lower GCS scores and longer lengths of stay compared to Group 3 (Table 3). Groups 1 and 2 also had a higher proportion of transferred patients and a trend toward significance in the proportion of patients with a new diagnosis of diabetes (*P* = 0.05). Group 1 had a higher proportion of cases with suspected or documented infection compared to Group 3, but no significant differences were observed between Groups 2 and 3. In Group 2, on presentation, 17 of 18 patients were tested for COVID-19 via polymerase chain reaction (PCR), and all tests were negative. In Group 3, all but two patients were tested for COVID-19, and only two had a documented positive result (both patients presented with new onset diabetes). The three remaining patients in Groups 2 and 3 who were not tested all presented early during the pandemic period (March 2020), and none had concerning symptoms of COVID-19. No significant differences were found between Groups 1 and 2 with respect to the rate of neuroradiological imaging, the time from presentation to initiation of hyperosmolar therapy, the time between the first and last hyperosmolar treatment, and the total number of hyperosmolar treatments. Only one patient in each suspected CABI group who underwent head CT had documented evidence of cerebral edema. No patient in Group 3 had neuroimaging. During both time periods, all patients with suspected CABI recovered without any documented neurologic deficits (all returned to “baseline” by the time of discharge).

No differences were found across the groups on any sociodemographic variables (Table 4).

**Figure 1:**
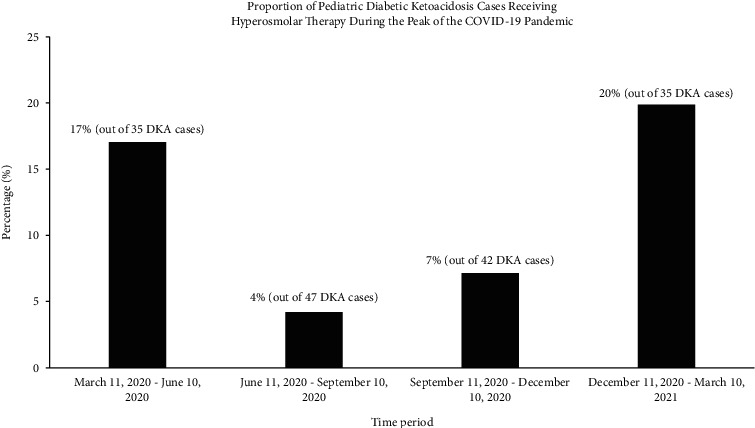
Proportion of DKA cases receiving hyperosmolar therapy (mannitol or hypertonic saline) for suspected CABI over the course of the year corresponding to the peak of the COVID-19 pandemic (March 11, 2020–March 10, 2021). The data are presented in three-month intervals. CABI, clinically apparent brain injury; DKA, diabetic ketoacidosis.

Figure 1 shows an initial decline in the proportion of DKA cases who received hyperosmolar therapy from 17.1% between March 11, 2020 and June 10, 2020 to 4.3% between June 11, 2020 and September 10, 2020, followed by a gradual increase up to 20% between December 11, 2020 and March 10, 2021. This overall pattern was similar to the trendline chart of new COVID-19 cases in Massachusetts over the same time period [14].

### 3.2. Discussion

We found an increased proportion of pediatric DKA cases who received hyperosmolar therapy during the peak of the COVID-19 pandemic compared to the three-year period preceding the pandemic. Furthermore, analysis of the proportions of patients who received hyperosmolar therapy in three-month blocks between March 2020 and March 2021 showed a pattern that overlapped with the Centers for Disease Control and Prevention's COVID Data Tracker for new cases of COVID-19 in Massachusetts over the same time period [14]. During times of surging COVID-19 infection rates and the associated lockdowns and restrictions, we speculate that there was anxiety about seeking medical care due to concern about risk of exposure to COVID-19. This may have contributed to delays in seeking medical care and resulted in higher rates of severe DKA at presentation and its associated complications. The BUN concentration is one of the previously identified predictors of CABI in DKA [11, 15–20]. We found the highest BUN concentration in patients with suspected CABI during the pandemic. Although this difference was only statistically significant between patients with and without suspected CABI during the peak of the pandemic, this finding suggests more severe dehydration as a result of a more protracted illness before presentation. This is further supported by the finding of a significantly higher BUN/creatinine ratio in Group 2 compared to the other two groups (a ratio >20 is suggestive of renal hypoperfusion, commonly due to dehydration). The overall increased incidence of suspected CABI during the pandemic could not be explained by new diabetes diagnosis versus established diabetes or patient transfer status. In addition, in our study, despite widespread testing via PCR, none of the patients who presented with suspected CABI had documented COVID-19 infection. Thus, COVID-19 infection could not explain the increased incidence of altered mental status leading to hyperosmolar treatment in this group.

Although several recent observational studies have shown an increased incidence of DKA in patients with T1DM during the COVID-19 pandemic, to our knowledge, no studies have reported a significant increase in the number of patients receiving hyperosmolar therapy compared to the prepandemic period. One potential explanation for this is the overall relatively low incidence of CABI. Severe, life-threatening brain injury complicates 0.3 to 1% of DKA events [10, 11, 21, 22]. If initiated in a timely fashion, management with hyperosmolar therapy and in some cases controlled hyperventilation [23–25] reduce rates of mortality and morbidity [26, 27]. However, although clinically apparent or overt brain injury is uncommon, neuroimaging studies have shown that some degree of cerebral edema is present in more than 50% of children with DKA [28], and evidence of neuronal injury is sometimes present without radiographic findings of cerebral edema [29]. This brain injury is often asymptomatic or associated with only minor mental status changes and is considered to be subclinical [28, 30–33].

As the effects of the pandemic, including the associated anxiety and hesitancy with regard to seeking medical care during surges in infection rates, may linger for several years, patients with T1DM, both newly diagnosed and established, may be at a persistently increased risk of developing severe metabolic decompensation that can lead to CABI. An awareness of this increased risk, especially with the possibility of novel vaccine-resistant variants leading to increases in COVID-19 cases or the emergence of new, highly transmissible infectious agents, is thus of paramount importance. This should motivate the design of national and community-level educational campaigns that target medical providers, parents, and others (e.g., teachers) involved in a child's care to be more vigilant about the signs and symptoms of new onset diabetes and DKA and promptly refer them to care. Such interventions have decreased the occurrence of DKA [34–36].

Additional potential implications of our findings should be considered. Historical data have shown that between 10% and 35% of survivors of CABI may have residual disabilities ranging from mild neurological impairment to a vegetative state in the most severe cases [10, 11, 21]. Although the latter finding is uncommon in the era of prompt recognition of the signs and symptoms of CABI and timely initiation of hyperosmolar therapy, both acute and long-term impairment of cognition and memory function, which have been found even in children with a history of DKA without evidence of overt cerebral edema, remain an important source of morbidity [37–45]. These neurocognitive deficits, however mild, may not only affect academic performance and future career prospects but also the child's ability to adhere to and participate in diabetes self-care, especially as they become more independent. None of our patients treated for suspected CABI had any documented overt neurologic sequelae (it must be noted that none had formal neuropsychological testing during admission) or evidence of worse short-term glycemic outcomes, based on the change in HbA1c between baseline and at least 12 months after presentation, compared to the group without suspected CABI. However, it is not clear at this time whether long-term executive functioning and glycemic control are affected by the presence of suspected CABI at presentation, inviting future investigations into this topic and requiring vigilant monitoring of these patients.

We recognize that there are several limitations to our study. By virtue of the retrospective chart review methodology, in many cases we could not obtain data on the precise signs and symptoms that led to the decision to administer hyperosmolar therapy, and the indication for initiating treatment likely varied among the different physicians managing the patients. Although all charts alluded to altered mental status, the diagnosis of CABI typically requires more rigorous criteria, which are usually not formally documented [46]. Thus, while we decided to include all patients who received hyperosmolar therapy for reported mental status changes, it is not clear whether all of these patients had CABI if a rigorous and systematic approach to diagnosis had been applied. It is certainly possible that different physicians may have varying thresholds for ordering hyperosmolar therapy, which could have been lowered during the pandemic. However, as there was no known formal change in the criteria for CABI diagnosis and treatment practices at our institution, the referring hospitals, or the critical care medical transport teams in recent years, it is reasonable to assume that the clinicians who made the decision to administer hyperosmolar therapy used similar clinical judgment both before and during the pandemic. This is also supported by the fact that the lowest documented GCS scores that often precede the administration of hyperosmolar therapy did not differ between the two suspected CABI groups. However, it is possible that patients with suspected CABI during the pandemic had more pronounced clinical features concerning for brain injury that were not captured by our medical record review, potentially explaining the increased proportion of these patients receiving hyperosmolar therapy compared to the prepandemic time period, despite similar biochemical characteristics and GCS scores between the two groups.

Another limitation is the relatively small sample sizes, particularly in the two suspected CABI groups. Consequently, where necessary, we conducted nonparametric testing. In addition, the presence of referral bias may have contributed to the statistically significant differences in the proportions of suspected CABI cases between the two time periods. Because the incidence of CABI is overall low, it is typically difficult to detect significant differences in studies performed at single institutions. It would be interesting to collaborate with other institutions across the nation and pool our data to determine whether our findings are in fact generalizable to other hospitals and regions. Despite these limitations, we hope that our study raises awareness of this uncommon but potentially life-threatening complication of pediatric DKA and leads to interventions that mitigate the risk of its occurrence especially during times of global fear and uncertainty as restrictions are lifted and novel COVID-19 variants appear or if new highly transmissible infectious agents emerge in the future.

## 4. Conclusions

Our study showed increased use of hyperosmolar therapy for suspected CABI in pediatric patients with DKA during the peak of the COVID-19 pandemic compared to the preceding three-year period, which was only significant among patients with newly diagnosed T1DM. During the pandemic, patients with suspected CABI had significantly higher BUN concentrations compared to patients without suspected CABI, suggesting more severe dehydration due to more protracted illness before presentation, presumably attributable to parental concern about exposure to COVID-19, resulting in delays in seeking medical care during surges in infection rates. These observations underscore the need for interventions to improve recognition of the symptoms and signs of diabetes and DKA among caregivers, healthcare workers, and others involved in the care of children and the crucial importance of immediate referral for medical care.

## Figures and Tables

**Table 1:** Incidence of suspected clinically apparent brain injury before and during the peak of the COVID-19 pandemic in pediatric patients presenting with diabetic ketoacidosis.

	Time period		Test statistic	*P* value
	Before pandemic	During pandemic		
*Total*				
Number of episodes of DKA (*n* = 503)	344	159		
Number of patients with suspected CABI (*n* = 36)	18	18		
Incidence of suspected CABI (7.3%)	5.2%	11.3%	*χ * ^2^ = 6.066	0.014
*New diabetes diagnosis versus established diabetes*				
New				
Number of episodes of DKA (*n* = 319)	216	103		
Number of patients with suspected CABI (*n* = 30)	15	15		
Incidence of suspected CABI (9.4%)	6.9%	14.6%	*χ * ^2^ = 4.751	0.029
Established				
Number of episodes of DKA (*n* = 184)	128	56		
Number of patients with suspected CABI (*n* = 6)	3	3		
Incidence of suspected CABI (3.3%)	2.3%	5.4%	Fisher's exact test	0.371

CABI, clinically apparent brain injury; DKA, diabetic ketoacidosis.

**Table 2:** Comparison of biochemical characteristics of patients presenting with suspected clinically apparent brain injury (CABI) before the COVID-19 pandemic, patients presenting with suspected CABI during the pandemic, and cases presenting with diabetic ketoacidosis but without suspected CABI during the pandemic.

Characteristics	Group			Test statistic	*P* value
	Suspected CABI before pandemic (group 1, *n* = 18)	Suspected CABI during pandemic (group 2, *n* = 18)	DKA without suspected CABI during pandemic (group 3, *n* = 141)		
*Biochemical variables * ^†^ * (mean* *±* *SD*^*‡*^)					
pH	6.89 ± 0.09	6.91 ± 0.13	7.14 ± 0.11	*H* = 61.032	<0.001^*∗*^
pCO2 (mmHg)	24.0 ± 6.0	21.1 ± 7.5	28.9 ± 7.5	*F* = 10.871	<0.001^*∗*^
Bicarbonate (mmol/L)	4.6 ± 1.7	4.4 ± 2.5	9.1 ± 3.4	*H* = 50.885	<0.001^*∗*^
BOHB (mmol/L)	9.4 ± 3.6	9.0 ± 4.0	7.3 ± 2.3	*H* = 4.419	0.110
Measured sodium (mmol/L)	134 ± 7	133 ± 5	133 ± 5	*H* = 1.667	0.43
Corrected sodium (mmol/L)	144 ± 7	142 ± 6	140 ± 6	*H* = 6.068	0.048^*∗∗*^
Potassium (mmol/L)	4.6 ± 1.1	4.4 ± 1.3	4.5 ± 0.8	*H* = 2.335	0.31
Anion gap (mmol/L)	33.3 ± 6.7	31.3 ± 5.4	28.1 ± 4.8	*H* = 13.333	<0.001^*∗*^
Measured osmolality (mOsm/kg)	356 ± 27	335 ± 22	326 ± 19	*H* = 9.429	0.009^*∗∗*^
Effective osmolality (mOsm/kg)	307 ± 17	302 ± 18	295 ± 13	*H* = 10.637	0.005^*∗∗*^
Glucose (mg/dL)	695 ± 275	658 ± 311	541 ± 217	*H* = 13.225	0.001^*∗∗*^
BUN (mg/dL)	19 ± 10	23 ± 10	16 ± 6	*H* = 12.381	0.002^*∗∗∗*^
Creatinine (mg/dL)	1.11 ± 0.45	0.90 ± 0.53	0.80 ± 0.38	*H* = 8.117	0.017^*∗∗*^
BUN/creatinine ratio	18.9 ± 9.2	28.5 ± 9.4	23.7 ± 15.1	*H* = 8.891	0.012^*∗∗∗∗*^
Initial HbA1c (%) (mmol/mol)	12.1 ± 1.8 (108.3 ± 19.6)	11.9 ± 2.2 (106.7 ± 23.9)	12.0 ± 2.3 (107.5 ± 24.7)	*H* = 0.086	0.96
Future HbA1c (%) (mmol/mol)	8.7 ± 2.1 (71.8 ± 23.3)	7.2 ± 0.7 (54.7 ± 7.4)	9.0 ± 3.0 (73.5 ± 37.2)	*H* = 4.550	0.10
HbA1c change from baseline^§^	−3.3 ± 2.1	−3.9 ± 2.0	−3.0 ± 3.6	*F* = 2.263^¶^	0.11

^†^On presentation, unless otherwise indicated. ^‡^Although means and standard deviations were reported for all variables, nonparametric testing was performed for comparison of mean ranks in the cases where data in at least one group were not normally distributed and and/or there were unequal variances across the groups. ^§^Adjusted for baseline HbA1c, interval between the two HbA1c measurements (in days), and new versus established diabetes status. ^¶^Due to unequal variances across groups, parameter estimates with robust standard error were calculated, with the following results: group 1–group 3: *t* *=* 1.063 and *P* *=* 0.290; group 2-group 3: *t* = −2.616 and *P* *=* 0.010. ^*∗*^On post hoc analysis, group 3 was significantly different from groups 1 and 2. ^*∗∗*^On post hoc analysis, group 3 was significantly different from group 1 but not group 2. ^*∗∗∗*^On post hoc analysis, group 3 was significantly different from group 2 but not group 1. ^*∗∗∗∗*^On post hoc analysis, group 2 was significantly different from groups 1 and 3. BOHB, beta-hydroxybutyrate; CABI, clinically apparent brain injury; DKA, diabetic ketoacidosis; SD, standard deviation; HbA1c, hemoglobin A1c.

**Table 3:** Comparison of clinical characteristics of patients presenting with suspected clinically apparent brain injury (CABI) before the COVID-19 pandemic, patients presenting with suspected CABI during the pandemic, and cases presenting with diabetic ketoacidosis but without suspected CABI during the pandemic.

Characteristics	Group			Test statistic	*P* value
	Suspected CABI before pandemic (group 1, *n* = 18)	Suspected CABI during pandemic (group 2, *n* = 18)	DKA without suspected CABI during pandemic (group 3, *n* = 141)		
*Clinical variables (mean* *±* *SD*^†^*or proportion)*					
New diagnosis of diabetes	83%	83%	62%	*χ * ^2^ = 5.989	0.05
Transferred from referring hospital	78%	72%	42%	*χ * ^2^ = 12.732	0.002^*∗*^
Initial GCS	13.3 ± 2.6	12.4 ± 3.6	14.9 ± 0.4	*H* = 34.489	<0.001^*∗*^
Lowest GCS	9.9 ± 3.7	10.1 ± 3.4	14.6 ± 1.0	*H* = 90.141	<0.001^*∗*^
Length of stay (days)^‡^	5.0 ± 2.8	4.4 ± 3.4	2.5 ± 1.4	*H* = 34.698	<0.001^*∗*^
Suspected or documented infection	33%	11%	9%	FHH exact test = 7.964	0.014^*∗∗*^
Rate of neuroradiological imaging	28%	17%	N/A	Fisher's exact test = 0.643	0.691
Time to suspected CABI development (mins)^§^	219 ± 168	140 ± 80	N/A	*U* = 123	0.23
Duration of treatment (mins)^¶^	308 ± 229	249 ± 239	N/A	*U* = 159	0.91
Number of interventions				Fisher's exact test = 0.190	0.91
1	56%	61%	N/A		
2	22%	17%	N/A		
3+	22%	22%	N/A		

^†^Although means and standard deviations were reported for all variables, nonparametric testing was performed for comparison of mean ranks in the cases where data in at least one group were not normally distributed and and/or there were unequal variances across the groups. ^‡^Two patients in group 3 who had lengths of stay of 123.9 and 63.1 days due to social issues or medical complexities unrelated to their diabetes diagnosis, respectively, were excluded from the analysis. ^§^Calculated as the time of presentation subtracted from the time of the first hyperosmolar therapy administration (in mins). ^¶^Calculated as the time difference between the first and last hyperosmolar treatment (in mins). ^*∗*^On posthoc analysis, group 3 was significantly different from groups 1 and 2. ^*∗∗*^On posthoc analysis, group 1 was significantly different from groups 2 and 3. CABI, clinically apparent brain injury; DKA, diabetic ketoacidosis; SD, standard deviation; FHH, Fisher-Freeman-Halton; GCS, Glasgow coma scale; N/A, not applicable.

**Table 4:** Comparison of sociodemographic characteristics of patients presenting with suspected clinically apparent brain injury (CABI) before the COVID-19 pandemic, patients presenting with suspected CABI during the pandemic, and cases presenting with diabetic ketoacidosis but without suspected CABI during the pandemic.

Characteristics	Group			Test statistic	*P* value
	Suspected CABI before pandemic (group 1, *n* = 18)	Suspected CABI during pandemic (group 2, *n* = 18)	DKA without suspected CABI during pandemic (group 3, *n* = 141)		
*Sociodemographic variables (mean ± SD * ^†^ * or proportion)*					
Age (years)	11.3 ± 4.4	10.9 ± 3.4	11.9 ± 4.2	*H* = 1.801	0.41
Female sex	39%	28%	44%	*χ * ^2^ = 1.791	0.41
Race/ethnicity				FHH exact test = 6.474	0.30
Black or African American	35%	18%	22%		
Hispanic or Latino	0%	0%	14%		
White	65%	82%	63%		
Other	0%	0%	3%		
Public insurance	39%	44%	42%	FHH exact test = 2.495	0.60
Caregiver health literacy score	4.9 ± 1.1	5.3 ± 1.4	5.1 ± 1.3	*H* = 2.077	0.35
Caregiver occupation code	1.4 ± 0.9	1.3 ± 1.2	1.7 ± 1.0	*H* = 3.349	0.18
Presence of intact family	61%	67%	58%	*χ * ^2^ = 0.506	0.78
Developmental/psychiatric concerns in child	56%	39%	29%	*χ * ^2^ = 5.420	0.07

^†^Although means and standard deviations were reported for all variables, nonparametric testing was performed for comparison of mean ranks in the cases where data in at least one group were not normally distributed and/or there were unequal variances across the groups. Abbreviations: CABI, clinically apparent brain injury; DKA, diabetic ketoacidosis; SD, standard deviation; FHH, Fisher-Freeman-Halton.

## Data Availability

Data available upon request from the corresponding author, Svetlana Azova (e-mail Svetlana.Azova@childrens.harvard.edu).
